# Structural Performance of a Hybrid FRP-Aluminum Modular Triangular Truss System Subjected to Various Loading Conditions

**DOI:** 10.1155/2014/615927

**Published:** 2014-08-28

**Authors:** Dongdong Zhang, Yaxin Huang, Qilin Zhao, Fei Li, Feng Li, Yifeng Gao

**Affiliations:** ^1^College of Field Engineering, PLA University of Science and Technology, Nanjing 210007, China; ^2^Institute of Logistics Engineering of PLA, Chongqing 400000, China

## Abstract

A novel hybrid FRP-aluminum truss system has been employed in a two-rut modular bridge superstructure composed of twin inverted triangular trusses. The actual flexural behavior of a one-rut truss has been previously investigated under the on-axis loading test; however, the structural performance of the one-rut truss subjected to an off-axis load is still not fully understood. In this paper, a geometrical linear finite element model is introduced and validated by the on-axis loading test; the structural performance of the one-rut truss subjected to off-axis load was numerically obtained; the dissimilarities of the structural performance between the two different loading cases are investigated in detail. The results indicated that (1) the structural behavior of the off-axis load differs from that of the on-axis load, and the off-axis load is the critical loading condition controlling the structural performance of the triangular truss; (2) under the off-axis load, the FRP trussed members and connectors bear certain out-of-plane bending moments and are subjected to a complicated stress state; and (3) the stress state of these members does not match that of the initial design, and optimization for the redesign of these members is needed, especially for the pretightened teeth connectors.

## 1. Introduction

Truss is an efficient structural form. According to the type of cross section and the number of chord members, trusses can be classified as plane trusses, triangular trusses, rectangular trusses, and other polygon trusses. Among these various cross section trusses, the triangular truss shows remarkable structural advantages over the conventional plane and rectangular trusses, such as the evident weight and material savings compared to the rectangular truss [1], the excellent torsional properties compared to the plane truss [2], and the lower number of joints compared to the rectangular truss [3]. The structural advantages and aesthetics of the triangular truss have long been recognized by structural engineers [2], and it has been widely used in civil infrastructures and aerospace engineering.

In civil infrastructures, the triangular truss has been employed on a limited basis for a number of structures such as roof truss girders, transmission towers, highway overhead sign structures, crane booms, portal frames, and offshore oil rig platform legs [2]. Common practices for the joint design of these structures are tubular welding joints and welded or bolted connections using gusset plates. The construction materials of these triangular trusses are mainly wood, steel, or aluminum materials. In addition to these applications, the triangular-truss configuration is popularly applied in aerospace engineering, such as the supporting structure of antenna array [4], solar array [5], space station [6] and airship [7–10], and the antennas of satellite [11, 12]. However, due to the critical weight control in aerospace engineering, their materials are mainly lightweight alloys or advance composite materials with high stiffness/strength-to-weight ratios. The joints are mainly welded or bolted connections, fusion joints or all-composite connectors [13, 14]. It is noteworthy that all of the triangular trusses mentioned are subjected mainly to continuously distributed loads or eccentric concentrated loads normal to the chord members or concentrated loads along the chord members, and the values of these loads are often nonheavy loads.

However, in bridge engineering, especially for heavy traffic loading, the instances in which a bridge truss of this nature has been designed and constructed are fewer than those in civil infrastructures and aerospace engineering. Actually, the first triangular-truss bridge was completed in 1930, and it was a through-type truss supporting two railway lines and constructed of lattice-box girders with diagonal bracing forming a triangular pattern [2]. A five-span triangular-truss bridge was built to carry monorail traffic. It was made up of two independent truss bridges, the single top chord of which supports the monorail [2]. It should be noted that the cross sections of these trusses are upright triangles, and the joints are mainly welded or bolted connections. With regard to the inverted triangular cross section truss, there are also some applications in bridges. For example, a deck-truss bridge has been built in Canada, and its two compression top chords support the floor beams and stringer for the deck, and a single lower chord is in tension [2]. A triangular cross section truss of modest span was designed as a deck-type bridge, which was composed of two lanes carrying a highway vehicle traffic loading [1]. In addition, an emergency truss bridge, named FB48, has been employed by the Swedish army. This emergency bridge was a deck-type bridge with twin triangular trusses and has a span of 48 m. Additionally, the analogous truss bridges FB56 and FB88 have been developed based on FB48 [15]. Today, the inverted triangular-truss continuation is more commonly applied as the truss girders that support the concrete deck in highway and railway bridges [16, 17]. These truss-girder bridges are often composed of twin inverted triangular trusses, which are fabricated entirely from unstiffened circular tubes, the connectors of which are welded tubular nodes. It is noted that the construction materials of these triangular trusses are mainly isotropic steel materials, and the instances in which composite materials with anisotropy have been used in these triangular-truss bridges are limited. A hybrid FRP-aluminum modular bridge has been designed by the authors, which was composed of twin inverted triangular trusses, and the modular structural units are connected by male jugs or female jaws based on a novel jointing system named the pretightened teeth connection (PTTC) [18]. It is noteworthy that the upper and lower chords are not integral due to its modular characteristic, unlike in the case of the existing continuous composite triangular trusses.

For the above-mentioned triangular-truss bridges, except for their dead weight, the loads directly subjected to the trusses are mainly the vehicle traffic load normal to the chord members. The chord members mainly bear compression and tension loads or are simultaneously combined with the bending moment. For the two-lane or multilane bridges, besides on-axis traffic load, these triangular trusses may also bear off-axis bending load, which has been recognized as the most critical load case and will lead to a certain torsional moment in the structure. In response to this problem, the literature [2] has mentioned that these trusses behave as excellent torsional properties because of their triangular cross sections; however, detailed studies have not been conducted in the literature. At present, according to the published literature, studies on the subject of the mechanical properties of these steel triangular-truss bridges subjected to off-axis loads are limited. With regard to the proposed modular composite triangular truss [18], the actual flexural behavior of a one-rut truss has been previously investigated under the on-axis bending loading test in the already published paper [18]. However, the structural performance of the truss under an off-axis loading case has still not been investigated, and this is the gap that this paper seeks to cover. Indeed, triangular trusses with differing structural configurations will exhibit respective torsional performances, and thus, the proposed modular composite triangular truss will show extraordinary structural behavior controlling the design of the FRP trussed members and connectors when subjected to an off-axis load.

In comparison with the paper that already published, the main attention in this work is to investigate the structural performance of the one-rut triangular truss subjected to off-axis load. In this paper, a simple description of the modular hybrid truss is presented. A geometrical linear finite element model is introduced and validated by the previous on-axis loading test. The structural performance of the one-rut triangular truss subjected to off-axis load was numerically obtained using the finite element model. The dissimilarities of the structural performance between the two different loading cases were then studied in detail.

## 2. Description of the Modular Triangular-Truss System

**Figure 1:**
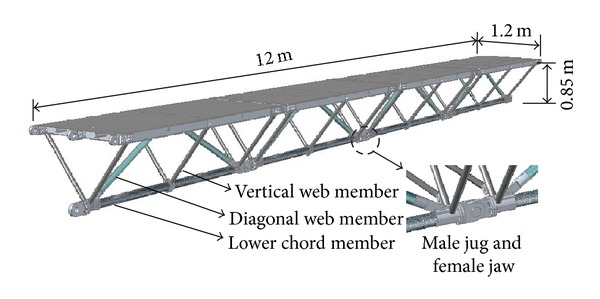
3D view of the one-rut triangular-truss system.

A hybrid FRP-aluminum space truss system was applied to a two-rut bridge superstructure, which enables a modular structural form to be erected and dismantled manually with individual structural units. It has a span length of 12 m and a total width of 3.2 m. The two-rut bridge is composed of twin triangular trusses, which are linked by transverse braces. Each truss was designed as an inverted triangular cross section with a 1.2 m width and 0.85 m depth (see Figure 1). Additionally, each rut is a deck-truss-type bridge structure and consists of an aluminum deck supported by FRP and aluminum trussed members. The four modular standard structural units are connected by male jugs and female jaws based on the pretightened teeth connection (PTTC). The total weight is approximately 6 kN for one rut and only 1.5 kN per unit. A 3D view of a one-rut triangular truss as designed is shown in Figure 1.

In the proposed triangular-truss system, pultruded FRP profiles and aluminum profiles for structural applications were selected for the trussed members (see Figure 1). A pultruded HFRP tube and GFRP tube were used for the lower chord members and diagonal web members, respectively. Here, the HFRP material consists of an admixture of E-glass fiber, carbon fiber, and basalt fiber embedded in an isophthalic vinyl ester resin. The wrought aluminum alloy 7A05 was selected for the vertical web members, bridge deck, and connectors. The mechanical properties of the composite materials used are listed in [18].

For the FRP trussed members, it is worth noting that all of these members were designed initially as pure tension-compression members and were only subjected to the axial direct forces in the preliminary design phase, in which the triangular truss was simplified as a plane truss model. In this analytical model, the triangular truss was simplified as a plane truss. The vertical and upper chord members were substituted for the two aluminum vertical web members and bridge deck, respectively. Then, the axial direct forces in the trussed members were calculated according to structural mechanics.

**Figure 2:**
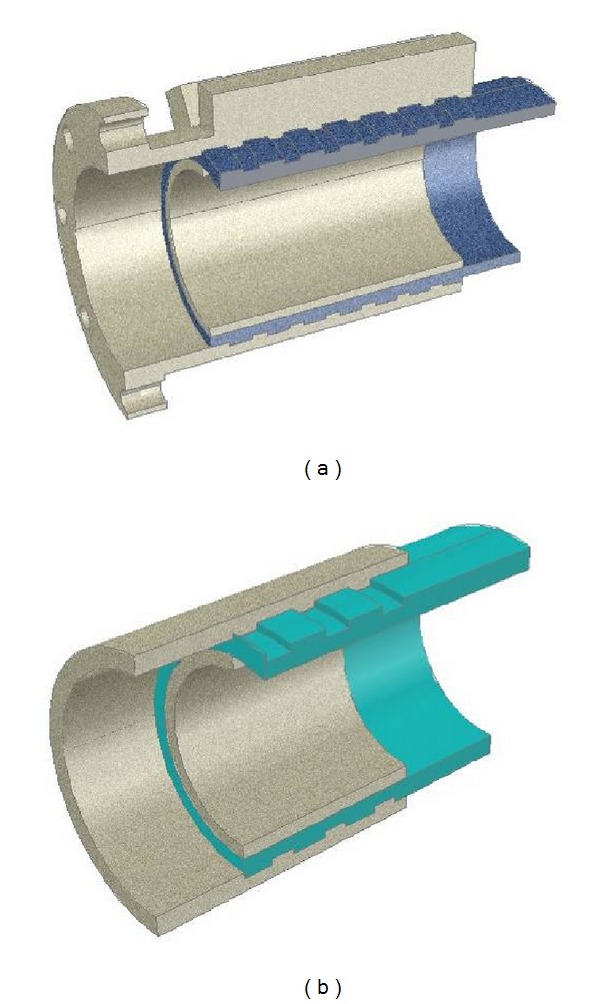
3D view of the composite tubular connection for (a) HFRP trussed members and (b) GFRP trussed members [18].

A novel joint system named the pretightened teeth connection (PTTC) is employed in the proposed modular triangular truss. Compared to the low connection efficiency attained using conventional composite-material connection techniques for FRP tubes, the pretightened teeth connection (PTTC) has higher connection efficiency [19]. The pretightened teeth connection involves a ring- or stripe-shaped tooth structure grooved at the connection end of the composite components and well-matched teeth on the aluminum piece connected with the composite (see Figure 2). After the composite tube is screwed into the inside of the external aluminum tube manually, the internal aluminum tube is compressed into the composite tube and the latter will be expanded because the external diameter of the internal aluminum tube is larger than the internal diameter of the composite tube. In this way, certain positive radial stresses (normal stresses) are exerted onto the composite teeth. The capacity to transfer a large external load is obtained because an interlaminar shear stress of the extrusion-type unidirectional fiber-reinforced composite material is higher than that of pure resin [20], and the radial compressive stress enhances the interlaminar shear strength.

It is worth stressing that this pretightened teeth connector was preliminarily designed as a pure tension-compression member; it was only subjected to the axial direct forces, and the bending moment was not considered or included. More information regarding the pretightened teeth connection, including its load-transfer mechanism, can be found in [19]. Based on the pretightened teeth connection, the junction of the aluminum constructional elements with the FRP trussed profiles can be easily realized. Welding technology was used for the junctions of aluminum profiles and HFRP/GFRP tubes for which the stresses are relatively small, whereas a flange joint was used for HFRP tubes with male jugs or female jaws, for which the stresses are much larger. The detailed design of the pretightened teeth connection and various junctions for the proposed triangular-truss system were presented in [18].

**Figure 3:**
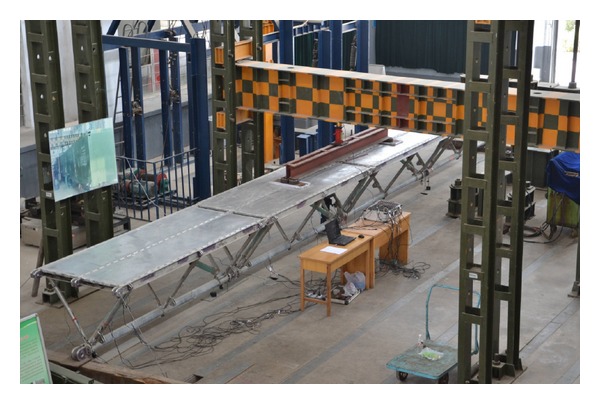
Single-span simply supported triangular truss and its on-axis four-point bending loading test setup.

Until now, four structural units of the prototype triangular-truss system have been fabricated by the factory. To understand the actual flexural behavior of the structure, these four prefabricated structural units were mounted as a simply supported structure and subjected to the on-axis four-point bending loading test, as shown in Figure 3. The experimental results showed the feasibility of the proposed hybrid triangular truss, which displayed a linear behavior under the ultimate limit of the loading level. Both the stiffness and strength were well within the design requirements, and the bridge features a sound structural flexural behavior in the on-axis loading condition.

However, because one of the connectors of the specimen was damaged at a large loading level over the service load in the on-axis loading condition, the relevant experimental study was not conducted under an off-axis load in [18]. The rupture of the connector was mainly due to the insufficient strength of the aluminum welding line in one of the GFRP diagonal web members. At present, all of the aluminum welding lines of the structural units are retreated in the factory.

## 3. Finite Element Model

In order to understand the structural performance of the specimen subjected to off-axis load, a geometrical linear finite element model was used. The finite element model (FEM) of the modular composite triangular truss was established using the general purpose finite element analysis software ANSYS 10.0. The finite element model is simply introduced as follows.

### 3.1. Modeling Process

To accurately simulate the proposed triangular-truss system, two types of elements (SHELL and BEAM elements) were employed to model the various structural members. The SHELL63 element was selected for the modeling of aluminum thin plate of the bridge deck, while the BEAM188 element was employed to simulate the crisscrossing “I” beams and trussed members, respectively. However, because it is difficult to precisely simulate the regions of the connectors located at both sides of the structural units, the connector and its conterminous chord members were all modeled using beam elements other than the solid elements. In the finite element model, the connectors were simplified as a segment of the corresponding chord members.

**Figure 4:**
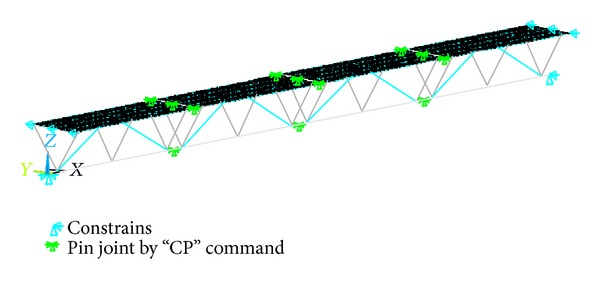
Finite element model of the one-rut triangular-truss structure.

The coordinate system was defined with the *x*-axis parallel to the lower chords, the *y*-axis parallel to the cross beams, and the *z*-axis parallel to the structure height. The origin of the coordinate system was at the node located at one side of the lower chord member, as shown in Figure 4. In this defined coordinate system, the displacement boundary conditions were set as follows: the nodes at *x* = 0, *y* = 0, and *z* = 0 were restrained in the *x*-, *y*-, and *z*-directions (pinned condition), and the nodes at *x* = 12 m, *y* = 0, and *z* = 0 were restrained in the *y*- and *z*-directions (roller condition). In addition, the nodes at *x* = 0, *y* = ±0.6 m, and *z* = 0.85 m and at *x* = 12 m, *y* = ±0.6 m, and *z* = 0.85 m were restrained in the *y-*direction to avoid lateral deflection. In addition, the pin joints were effectively realized by defining a set of coupled degrees of freedom using the command “CP.”

In this linear anisotropic material model, the buckling issue of the compressive members was not considered. The elasticity modulus and Poisson's ratio used in the finite element model were provided by the manufacturer and are summarized in Table 1. In addition, the geometrical nonlinearity was also not considered in the finite element model. The finite element model of the entire specimen is shown in Figure 4. The detailed finite element model was established in [18].

### 3.2. Loading Cases

**Figure 5:**
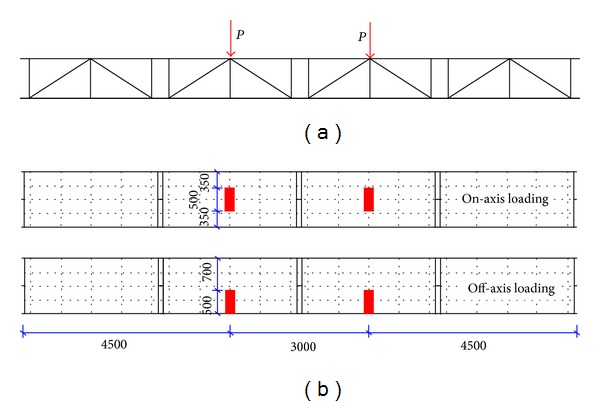
Two different loading configurations in the finite element model. (a) Side view and (b) top view (dimensions in mm).

Differ from [18], in the finite element model (FEM), two loading cases were simulated according to the practical traffic loading conditions, referred to as the on-axis loading condition and the off-axis loading condition, as shown in Figure 5.

During the loading process for each case, the corresponding uniformly distributed wheel load was transformed into point loads acting on each node in the loading area of the thin plate. The numerical simulations were performed with eight loading levels (2P = 10, 20, 32, 40, 52, 60, 70, and 75 kN). The dead weight was not considered in this finite element analysis.

The stress state in certain FRP trussed members and the displacement at the midspan of the lower chord member were abstracted, respectively. The numerical results of the on-axis loading model are presented in Section 4 and are compared with experimental results to further validate the finite element model; the comparison of the on-axis loading model and the off-axis loading model is presented in Section 5 to obtain the dissimilarity of the structural performances of the triangular truss under the two different loading conditions.

## 4. Verification of the Finite Element Model by Experiment

In this section, based on the on-axis four-point loading test that was conducted in previous work [18], the validation of the established finite element model (FEM) was further performed by comparing the numerical solutions with the experimental results, including the vertical displacement at the midspan of the lower chord member and the internal axial direct forces in some FRP trussed members.

### 4.1. Displacement at the Midspan

For this hybrid triangular-truss system, the deflection is an important factor affecting the normal use of the structure, considering the requirement of the guidelines. In the validation, the vertical displacement was compared for the numerical values and the experimental results. Indeed, in the on-axis four-point bending loading test [18], two loading steps were applied to the specimen: from 0 kN to 56 kN with an interval of 4.0 kN in the first step and then from 56 kN to 75 kN with an interval of 2.0 kN in the second step. However, in this paper, only eight loading levels were selected and compared with the numerical results, and they are 0, 10, 20, 32, 40, 52, 60, 70, and 75 kN.

**Figure 6:**
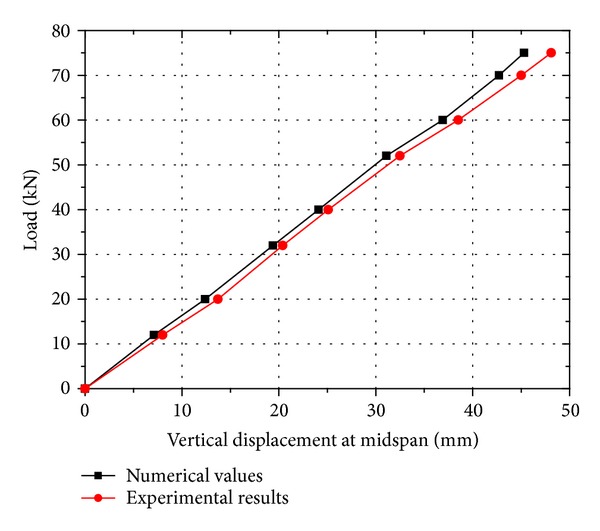
Comparison of the vertical displacement between the numerical values and the experimental results.

Figure 6 shows the comparison of two load-displacement curves obtained from the finite element model and experiments subjected to an on-axis load. It can be found that the two curves are linear and notably close to each other. The maximum difference between the two curves is less than 1.5%. Thus, the finite element model can be used to predict the displacement of the proposed triangular-truss system with satisfactory accuracy.

### 4.2. Internal Axial Direct Forces in the FRP Trussed Members

**Figure 7:**
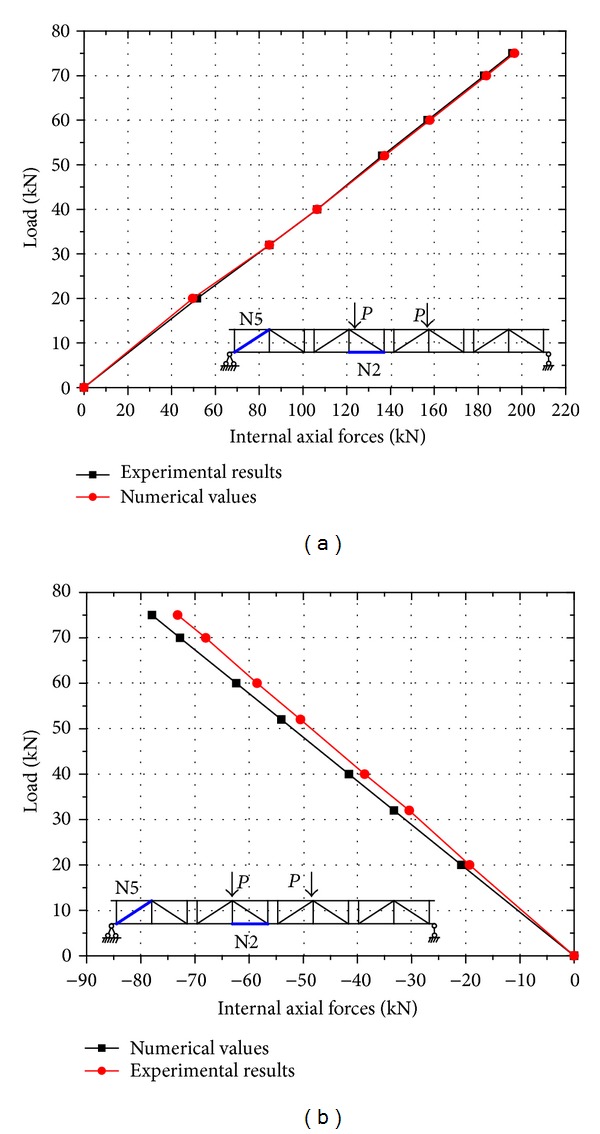
Comparison of the internal axial forces in the trussed members between FEA and test. (a) N2 and (b) N5.

For the internal axial direct forces, only two representative FRP trussed members (N2 and N5) were compared between the numerical solutions and the experimental results (see Figure 7). It is noted that, in [18], only the internal axial direct forces of the maximum load level were compared; however, in this work, about seven load levels were calculated and compared to obtain a more reliable verification result. As shown in Figure 7, the internal axial direct forces in the compared trussed members are very similar to the two curves, especially for the N2 trussed member. In member N5, the discrepancy of the two curves is very small, and the maximum difference is approximately 6.1%. Thus, the finite element model is also an available model in terms of calculating the internal forces in the FRP trussed members of the hybrid triangular-truss system.

In general, the finite element model (FEM) can accurately predict mechanical behaviors such as the deflections and internal forces of the proposed hybrid triangular-truss system. The modeling techniques can be used to analyze the structural performance of the specimen under the two different loading conditions.

## 5. Numerical Results of the Two Different Loading Conditions and Their Comparison

In this section, the numerical results under the two different loading conditions are presented and compared to obtain the dissimilarities of the structural performance, including the vertical and horizontal displacement at the midspan of the lower chord member, the deformation of the HFRP lower chord member, and the stress state of FRP trussed members and the connectors.

### 5.1. Displacement and Deformation

**Figure 8:**
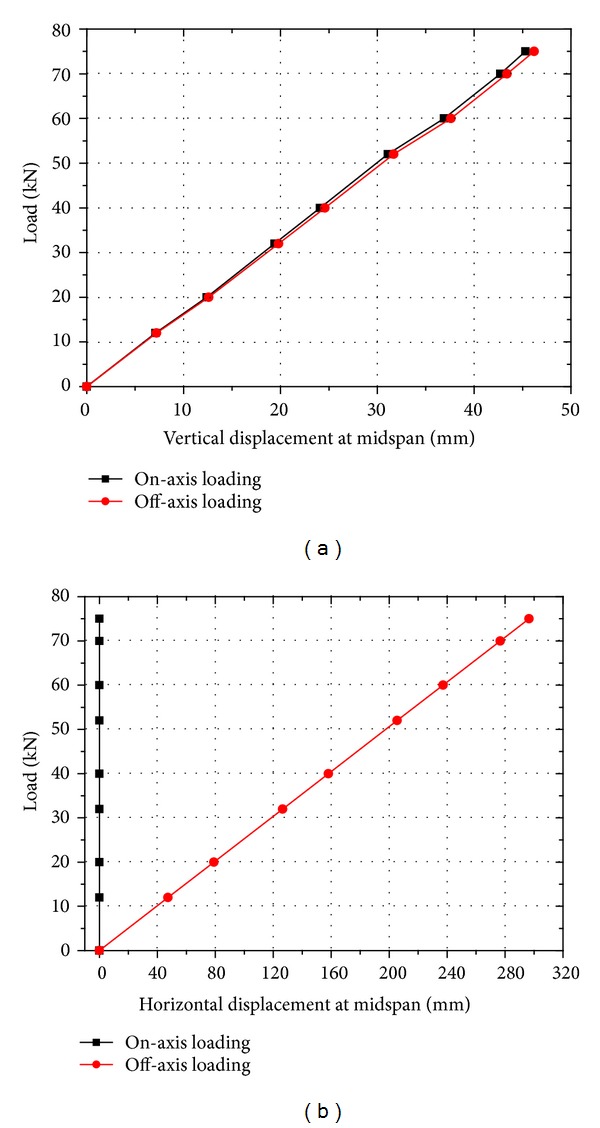
Comparison of the displacement at the midspan of the lower chord member between the on-axis load and the off-axis load. (a) Vertical displacement and (b) horizontal displacement.

Figure 8 shows the comparison of the displacement at the midspan of the lower chord member of the structure between the two different loading cases. In Figure 8, the vertical displacement and the horizontal displacement were compared, respectively. It can be found that, for the vertical displacement, the two loading-displacement curves are both linear and notably close to each other (see Figure 8(a)). The maximum difference between the two curves is less than 1.9%. That is, the off-axis loading condition will not alter the vertical displacement of the proposed triangular-truss system for the on-axis loading case. The obtained load-displacement curves have a slope of 1.546 × 10^6^ N/m, which is an indication of the integral structural stiffness (flexural stiffness) of the proposed hybrid triangular-truss system. In addition, the maximum vertical displacement under the on-axis load and the off-axis load is 45.3 mm and 46.2 mm, respectively, and both are below the admissible deflection limit with a value of 80 mm (L/150) recommended by the military bridge design code GJB 1162-91 [21]. The structure is still in the elastic regime at the ultimate limit of the loading level.

However, for the horizontal displacement, it clearly shows that the values vary dramatically between the on-axis loading and the off-axis loading curves (Figure 8(b)). The horizontal displacement under each on-axis loading level is approximately 0 mm; however, the maximum horizontal displacement can reach as high as 296 mm at a loading level of 75 kN (about 1/4 of the width of structure). In the off-axis loading condition, the large horizontal displacements are mainly caused by the torsional moment. For the horizontal displacement, the admissible limit is not recommended by the military bridge design code GJB 1162-91 [1].

**Figure 9:**
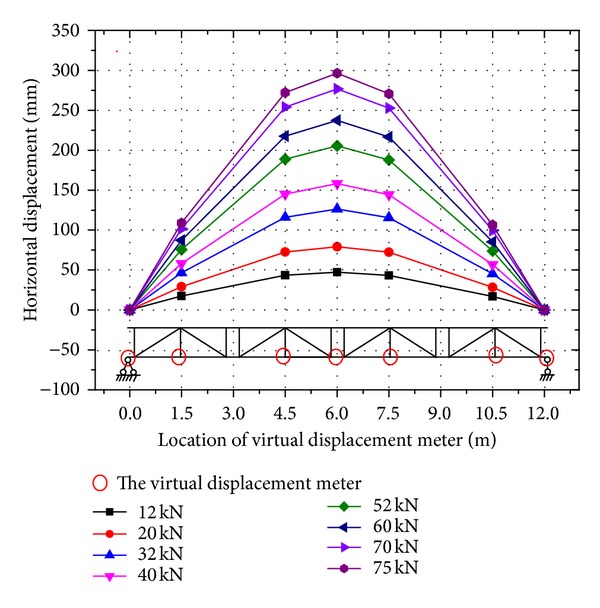
Variation in the overall horizontal displacement of the HFRP lower chord member with increasing applied off-axis load P.

Figure 9 shows the horizontal deformed shape of the HFRP lower chord members at various off-axis loading levels. It is noted that the measured deformed shape appears uniformly with an increasing applied load, and the shape is symmetrical about the span center. The displacement is greatest at the midspan among the virtual gauging points at each loading level. The horizontal deformed shape of the lower chord members under various on-axis loading levels is not plotted because the values are all 0 mm. Therefore, the horizontal displacement is the main dissimilarity of the proposed triangular truss under the two different loading cases.

### 5.2. Stress State in the FRP Trussed Members and the Connectors

In this section, the stress states in the FRP trussed members and the connectors were compared between the on-axis loading and the off-axis loading, respectively. The stress states include the axial direct stress, bending stress, and superimposed stress in the *X*-*Z* plane and the *X*-*Y* plane. Here, the superimposed stress is the sum of the axial direct stress and the bending stress. The coordinate system was defined with the *x*-axis parallel to the lower chords, the *y*-axis parallel to the cross beams, and the *z*-axis parallel to the structure height. Namely, the stresses in the *X*-*Z* plane and the *X*-*Y* plane are referred to as the in-plane (vertical plane along the truss) and the out-of-plane (horizontal plane along the truss) stresses, respectively. It is noted that all the numerical stress states in this section were calculated at the maximum load level of 75 kN.

Figures 11–15 show the comparison of the stress contours in the HFRP lower chord members of the two different loading models at the maximum loading level. All of the stress contours were directly extracted from the finite element models. Due to space confinements, the stress contours of the GFRP diagonal web members are not presented in this paper because the regularity of the comparison results is the same as that of the HFRP lower chord members described in the following paragraphs. As displayed in Figure 11, the lower chord members are subjected to certain axial direct stresses, and the maximum value is approximately 79 MPa, which is less than the material strength. The distribution and values of the axial direct stress in the lower chord member are similar to the two different loading models.

**Figure 10:**
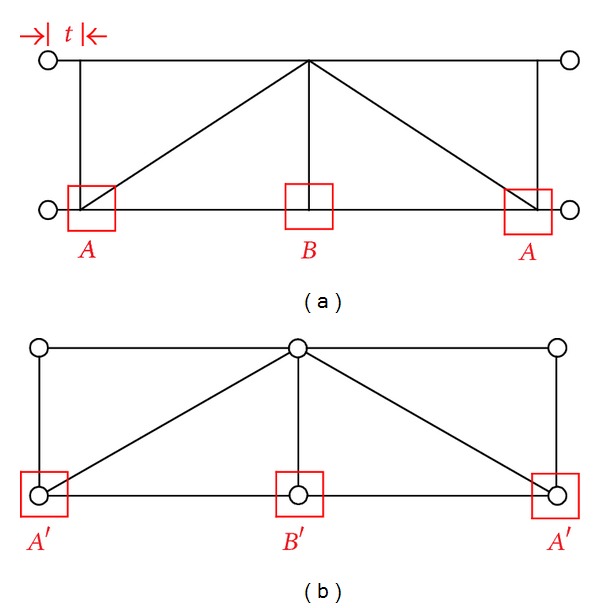
Comparison of the jointing nodes. (a) The actual triangular truss and (b) the ideal simplified plane truss.

Figure 12 shows the bending stress contours in the *X*-*Z* plane of the HFRP lower chord members. It can be found that there were certain bending stresses in the lower chord members. Additionally, the maximum stresses are mainly distributed at the two sides of the lower chord member for each structural unit (the two sides are marked as box “*A*” in Figure 10(a)), and the maximum value along the overall lower chord member is approximately −82.8 MPa for both loading cases. It is noteworthy that this stress state does not match that of the initial design, in which the lower chord members were only subjected to axial direct forces and were all pure tension-compression members. This is mainly attributed to the dissimilarities of the joints between the actual truss and the ideal simplified plane truss, as shown in Figure 10. In the ideal simplified plane truss used in the initial design, the nodes* A*′ and* B*′ are all hinge-jointed nodes; however, in the actual space truss, the nodes *A* and *B* are rigid-jointed nodes. In addition, the “*t*” values are 0 mm and 185 mm in the simplified plane truss model and the actual space triangular truss, respectively (see Figure 10). That is, the three vertical web members are directly and rigidly joined to the lower chord member other than the connectors. When the truss is subjected to an off-axis load, part of the external loads are transferred to the vertical web members, and the vertical component of the forces in these vertical web members are then subjected to the lower chord members. Thus, the lower chord member bears a certain bending moment in the *X*-*Z* plane.

In regard to the comparison of the two different loading models, the distributions of the bending stresses in the *X*-*Z* plane are nearly identical, and the difference of the maximum value is only 0.63%. Namely, the off-axis loading condition will not change the *X*-*Z* plane bending stress of the proposed triangular truss under an on-axis loading condition. This is mainly because the vertical components of the applied load in the *X*-*Z* plane are equal to each other for the two different loading cases, and thus, the bending moments in the *X*-*Z* plane are identical.

However, for the bending stress in the *X*-*Y* plane, there is a large dissimilarity between the two different loading models, as shown in Figure 13. In the on-axis loading condition, the bending stresses along the lower chord members are all equal to approximately 0 MPa (Figure 13(a)); in contrast, in the off-axis loading condition, the maximum value is approximately 110.2 MPa (Figure 13(b)). The maximum stresses are mainly distributed at the two sides of the lower chord member for each structural unit (the two sides are marked as box “*A*” in Figure 10(a)), as shown in Figure 13(b). The HFRP lower chord members are subjected to bending moments both in the *X*-*Y* plane and in the *X*-*Z* plane under the off-axis loading, and the maximum stress in the *X*-*Y* plane (Figure 13(b)) was approximately 3.336 times that in the *X*-*Z* plane (Figure 12(b)). Therefore, compared to the on-axis loading, the off-axis loading has a greater influence on the *X*-*Y* plane bending moment in the HFRP lower chord members. This is mainly because, under an off-axis load, part of the external load is transferred to the vertical web members, and the horizontal components of the forces in these vertical web members are then subjected to the lower chord member. In addition, the truss bears a torsional moment under an off-axis load, and the truss exhibits a torsionally deformed shape that enlarges the horizontal component of the forces. Therefore, the lower chord members bear certain bending moments in the *X*-*Y* plane. From another point of view, under an off-axis traffic load, part of the torsional moment of the triangular truss is resisted by the horizontal bending deformation of the lower chord members.

In addition to the aforementioned individual axial direct stress and the bending stress contours, the superimposed stress contours were also extracted and analyzed. Figures 14 and 15 show the superimposed stress contours of the HFRP lower chord members in the *X*-*Z* and *X*-*Y* planes, respectively. In Figure 14, the distributions of the superimposed stress in the *X*-*Z* plane are nearly identical, and the values are equal to each other between the two finite element models. However, in the *X*-*Y* plane, there is a large dissimilarity for either the distribution or the values between the two different loading models; additionally, the maximum *X*-*Y* plane stress in the off-axis loading case is approximately 2.134 times that in the on-axis loading case, as shown in Figure 15. In addition, under the off-axis loading, the maximum superimposed stress in the *X*-*Y* plane (Figure 15(b)) is approximately 2.073 times that in the *X*-*Z* plane (Figure 14(b)); in contrast, under on-axial loading, the maximum superimposed stress in the *X*-*Y* plane (Figure 15(a)) is equal to that in the *X*-*Z* plane (Figure 14(a)). That is, for the HFRP lower chord members, the off-axis load only influences the superimposed stress in the *X*-*Y* plane other than in the *X*-*Z* plane.

Indeed, the aforementioned superimposed stress is a sum of the axial direct stress and the bending stress. That is, the superimposed stress in the *X*-*Z* plane is the sum of the axial direct stress and the bending stress in the *X*-*Z* plane, and the superimposed stress in the *X*-*Y* plane is the sum of the axial direct stress and the bending stress in the *X*-*Y* plane. Thus, the distribution of the axial direct stress and the bending stress determine the distribution of the superimposed stress. Additionally, the regularity of the comparison results of the bending stress is identical to that of the superimposed stress.

**Figure 11:**
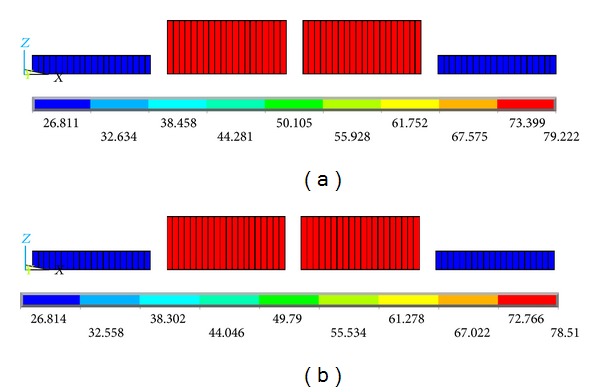
Contours of the internal direct axial stress in the HFRP lower chord members. (a) On-axis loading case and (b) off-axis loading case (units in MPa).

**Figure 12:**
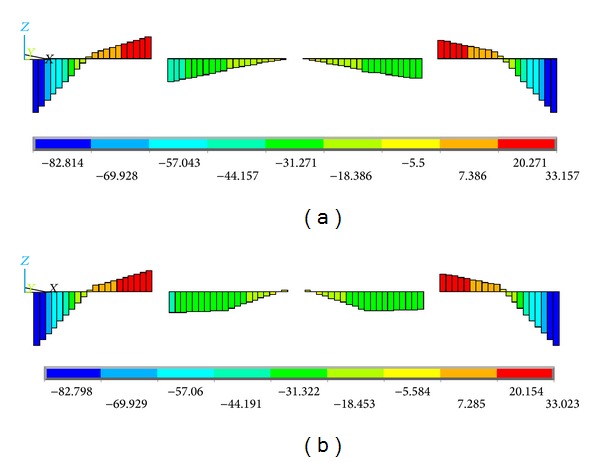
Contours of the *X*-*Z* plane bending stress of the HFRP lower chord members. (a) On-axis loading case and (b) off-axis loading case (units in MPa).

**Figure 13:**
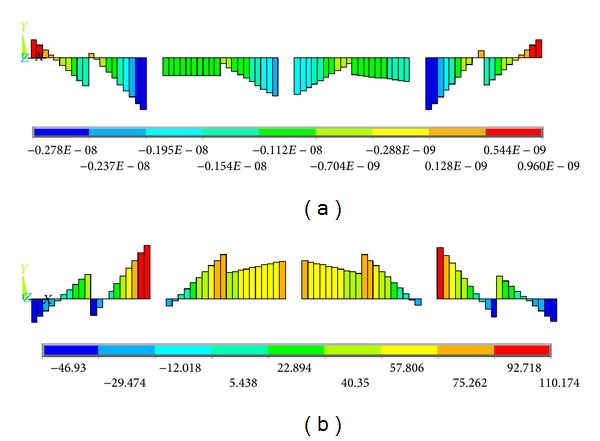
Contours of the *X*-*Y* plane bending stress of the HFRP lower chord members. (a) On-axis loading case and (b) off-axis loading case (units in MPa).

**Figure 14:**
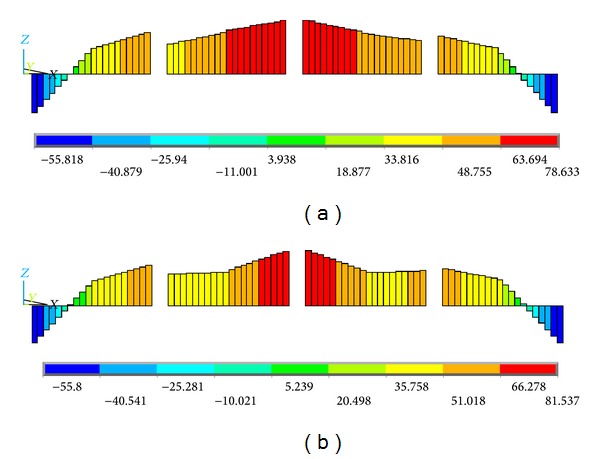
Contours of the *X*-*Z* plane superimposed stress of the HFRP lower chord members. (a) On-axis loading case and (b) off-axis loading case (units in MPa).

**Figure 15:**
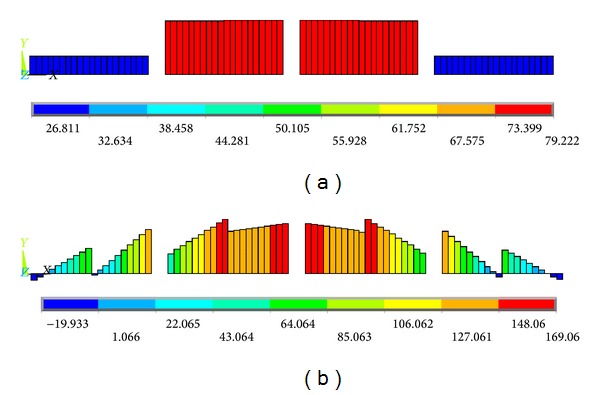
Contours of the *X*-*Y* plane superimposed stress of the HFRP lower chord members. (a) On-axis loading case and (b) off-axis loading case (units in MPa).

Combining Figures 14 and 15 with Figures 11–13, the proportion of the axial direct stress and the bending stress accounting for the maximum superimposed stress in the lower chord members, respectively, can be obtained. The results are summarized in Table 2, the data of which were obtained at the maximum load level of 75 kN. As shown in Table 2, in the *X*-*Z* plane, the bending stress possesses a small part in the superimposed stress, and the axial direct stress accounts for almost the entire part of either the on-axis loading model or the off-axis loading model. In addition, the same stress results exist in the *X*-*Y* plane under the on-axis loading. Indeed, because the lower chord members were designed according to the maximum superimposed stress, and the axial direct stress accounts for almost the entire maximum superimposed stress, this stress state does match that of the initial design, in which the lower chord members are only subjected to pure axial direct stress.

However, for the bending stress in the *X*-*Y* plane under off-axis loading, the result is significantly different: the ratios of the axial direct stress and the bending stress are 46.44% and 53.56%, respectively. The bending stress and the axial direct stress play nearly equivalent roles in the maximum superimposed stress. This stress state does not match that of the initial design, in which the lower chord members are only subjected to the axial direct forces and were pure tension-compression members. In general, this shows that the off-axis loading condition also plays a great role in the *X*-*Y* plane superimposed stress.

For the connectors joining the lower chord members, the value and proportion of the axial direct stress and the bending stress accounting for the maximum superimposed stress were also extracted. The results are summarized in Table 3, the data of which were also obtained at the maximum load level of 75 kN. The superimposed stress contours are not presented here due to the limitation of coverage. As shown in Table 3, in the *X*-*Y* plane, the axial direct stress possesses 100% of the superimposed stress in the on-axis loading case; however, under an off-axis load, the bending stress and the axial direct stress play nearly equivalent roles in the maximum superimposed stress. The connectors bear certain bending stresses in the *X*-*Y* plane mainly because of the torsion moment of the truss subjected to an off-axis load. In the *X*-*Z* plane, the bending stress and the axial direct stress also play nearly equivalent roles in the maximum superimposed stress under either an on-axis load or an off-axis load. The certain bending moment in the *X*-*Z* plane is mainly attributed to the dissimilarities of the joints between the actual truss and the ideal simplified plane truss (see Figure 10), as analyzed in paragraph three of Section 5.2. Generally, both the axial direct forces and the local bending moment exist in the connectors under an off-axis loading, and this stress state does not match that of the initial design in which the connectors are only subjected to tension or compression forces.

In addition, in the *X*-*Z* plane, it shows that the superimposed stress in the off-axis loading case is equal to that in the on-axis loading case; in contrast, in the *X*-*Y* plane, the superimposed stress in the off-axis loading case is approximately 1.944 times that in the on-axis loading case. Namely, the off-axis loading condition plays a critical role on the superimposed stress of the connectors.

In summary, the off-axis loading case plays a critical role in controlling the bending stress and the superimposed stress out of the plane in the lower chord members and connectors, respectively. When subjected to an off-axis load, there are certain out-of-plane bending moments in the lower chord members and the connectors, which is mainly due to the torsional moment of the truss along with the specific node configuration. When subjected to both an off-axis load and an off-axis load, the lower chord members and connectors bear a certain in-plane bending moment, which is mainly attributed to the specific node configuration of the truss. The stress state in these members does not match that of the initial design, in which the lower chord members and connectors are only subjected to the axial direct forces and were pure tension-compression members. In addition, the superimposed stress under an off-axis load is much larger than that under an on-axis load. The triangular truss should be designed critically according to the off-axis loading condition other than the on-axis loading condition.

### 5.3. Discussion

For the HFRP lower chord members, the off-axis load is the critical loading condition controlling the *X*-*Y* plane stress. In the *X*-*Y* plane, the bending stress and the axial direct stress account for nearly equivalent parts of the maximum superimposed stress under the off-axis load, and this stress state does not match that of the initial design, in which the lower chord members are only subjected to pure axial direct forces. Thus, these HFRP lower chord members cannot simply be designed according to the pure tension-compression member, and the bending moment should be included in the initial design. Moreover, the results show that the maximum bending stress in the *X*-*Y* plane is approximately 3.336 times that in the *X*-*Z* plane under an off-axis load, and the included bending moment should select the maximum value in the *X*-*Y* plane under an off-axis load.

Indeed, if the isotropic aluminum material is used for these lower chord members, the bending problem cannot be highlighted. However, it should be given significant consideration for anisotropic composite materials because the interlaminar shear stress of the extrusion-type unidirectional fiber-reinforced composite materials is low. When subjected to certain bending and shear stresses, the HFRP lower chord members will bear a complicated and harmful stress state that may cause the cracks of the FRP composite tube. Thus, the HFRP lower chord members should be designed critically according to the structural properties of the proposed triangular truss subjected to an off-axis load.

**Figure 16:**
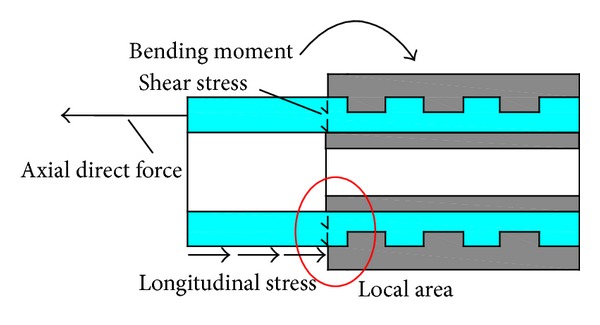
Diagram of the loading state in the pretightened teeth connector.

For the connectors, the bending stress and the axial direct stress also account for a nearly equivalent portion of the maximum superimposed stress in both the *X*-*Y* and *X*-*Z* planes under an off-axis load; and the same results apply to the stress state in the *X*-*Z* plane under an on-axis load. Namely, in addition to the axial direct force, the connectors bear a certain local bending moment in all of the various loading conditions (see Figure 16). The connectors are subjected to a complicated stress state, which does not match the pure tension-compression state in the initial design. In addition, the *X*-*Y* plane superimposed stress in the off-axis loading case is approximately 1.944 times that in the on-axis loading case. Namely, the off-axis loading condition plays a critical role in controlling the superimposed stress of the connectors.

When subjected to a local bending moment, the connector will bear certain additional longitudinal stresses along the connector. Indeed, in the connector, there are also certain shear forces that will cause local shear stress at the local area (the change position) of the cross section (see Figure 16). Because of the low interlaminar shear stress of the extrusion-type unidirectional fiber-reinforced composite materials, the composite tube of the connector may cause longitudinal cracks under the additional longitudinal stress, along with local shear damage under local shear stress. Thus, these connectors based on the pretightened teeth connection cannot simply be designed as pure tension-compression members. Only if the axial direct force, the local bending moment, and the shear forces are all included in the initial design, the connectors enable a larger bearing capacity and exhibit better structural properties. The pretightened teeth connector will be redesigned for this modular truss to obtain a larger bearing capacity and the structural properties under a complicated stress state will be investigated in detail in forthcoming works.

## 6. Conclusions

A hybrid FRP-aluminum modular truss system was introduced, which is composed of twin triangular trusses and is connected by male jugs and female jaws based on the pretightened teeth connection technique. The structural performance of its one-rut triangular truss subjected to an off-axis load was obtained numerically using the finite element model, which has been validated by the on-axis loading test. The dissimilarities of the structural performance between the two different loading cases were studied in detail. The following conclusions were drawn.

1. The structural behavior of the off-axis load differs much from that of the on-axis load, and the off-axis loading case plays a critical role in controlling the structural performance of the triangular truss. The triangular truss should be designed critically according to the off-axis loading condition other than the on-axis loading condition.
2. For the vertical displacement, two loading-displacement curves are both linear and notably close to each other under two different loading cases, and the maximum difference between the two curves is less than 1.9%. However, for the horizontal displacement, the values vary dramatically between the on-axis loading and the off-axis loading curves. The large horizontal displacements are mainly caused by the torsional moment of the truss subjected to an off-axis load.
3. For the internal force in the FRP trussed members (or the pretightened teeth connector), the only dissimilarity between the two different loading cases is the out-of-plane bending stress. The off-axis loading condition plays a critical role in controlling the out-of-plane bending stress and the superimposed stress. In addition, under an off-axis load, the maximum out-of-plane bending stress and superimposed stress is much larger than the corresponding in-plane stresses.
4. For the FRP trussed members, the in-plane bending stress possesses a small part of the superimposed stress, and the axial direct stress accounts for almost the entire part in either the on-axis loading case or the off-axis loading case. However, due to the torsional moment along with specific node configuration, these members bear certain out-of-plane bending stresses under off-axial loads. The out-of-plane bending stresses and the axial direct stresses account for a nearly equivalent portion of the maximum superimposed stress. This stress state does not match that of the initial design, in which the lower chord member was designed as a pure tension-compression member. Therefore, these HFRP lower chord members should be redesigned critically according to the structural properties under off-axis loads.
5. Due to the specific node configuration, the pretightened teeth connectors bear a certain in-plane bending moment under both loading cases; in addition, due to the torsional moment along with specific node configuration, they also bear a certain out-of-plane bending moment under an off-axis load. These bending stress and axial direct stress account for a nearly equivalent proportion of the maximum superimposed stress. Namely, both axial direct forces and the local bending moment exist in the connectors and the connectors are subjected to a complicated stress state. The stress state does not match up to that of the initial design using a simplified plane truss model, in which they were only subjected to pure tension-compression forces. Thus, these connectors should also be redesigned and optimized for this type of modular triangular truss.

## Figures and Tables

**Table 1:** Mechanical properties of all materials used in the finite element model.

Materials & members	Modulus of elasticity (GPa)	Poisson's ratio
HFRP members	*E*_1_ = 61.6, *E*_2_ = *E*_3_ = 8.3; *G*_12_ = *G*_13_ = 5.1, *G*_23_ = 4.6	*ν*_12_ = *ν*_13_ = 0.31, *ν*_23_ = 0.34
GFRP members	*E*_1_ = 31.5, *E*_2_ = *E*_3_ = 7.2; *G*_12_ = *G*_13_ = 4.4, *G*_23_ = 4.1	*ν*_12_ = *ν*_13_ = 0.28, *ν*_23_ = 0.33
Al alloy members	*E* = 60	*ν* = 0.33

**Table 2:** Comparison of the maximum superimposed stress with the corresponding individual stresses in the lower chord members at the maximum load level of 75 kN.

Comparison of the maximum superimposed stress in the lower chord members	In *X*-*Z* plane			In *X*-*Y* plane		
	Superimposed stress	Axial direct stress	Bending stress	Superimposed stress	Axial direct stress	Bending stress
On-axis load						
Value (MPa)	78.63	79.22	−0.59	79.22	79.22	0
Ratio (%)	/	100.75	−0.75	/	100	0
Off-axis load						
Value (MPa)	81.54	78.51	3.03	169.06	78.51	90.55
Ratio (%)	/	96.28	3.72	/	46.44	53.56
Off-axis value to on-axis value ratio	1.037	0.991	/	2.134	0.991	/

**Table 3:** Comparison of the maximum superimposed stress in the connectors at the maximum load level of 75 kN.

Comparison of the maximum superimposed stress in the connectors	In *X*-*Z* plane			In *X*-*Y* plane		
	Superimposed stress	Axial direct stress	Bending stress	Superimposed stress	Axial direct stress	Bending stress
On-axis load						
Value (MPa)	114.15	59.50	54.65	82.35	82.35	0
Ratio (%)	/	52.12	47.88	/	100	0
Off-axis load						
Value (MPa)	114.15	59.26	54.89	160.08	82.35	77.73
Ratio (%)	/	51.91	48.09	/	51.44	48.56
Off-axis value to on-axis value ratio	1.000	0.996	/	1.944	1.000	/
